# Transcriptome Analysis of Tomato Leaf Spot Pathogen *Fusarium proliferatum*: *De novo* Assembly, Expression Profiling, and Identification of Candidate Effectors

**DOI:** 10.3390/ijms19010031

**Published:** 2017-12-22

**Authors:** Meiling Gao, Siyu Yao, Yang Liu, Haining Yu, Pinsan Xu, Wenhui Sun, Zhongji Pu, Hongman Hou, Yongming Bao

**Affiliations:** 1 School of Life Science and Biotechnology, Dalian University of Technology, Dalian 116024, China; gaomeiling@mail.dlut.edu.cn (M.G.); yuhaining@dlut.edu.cn (H.Y.); xupinsan@dlut.edu.cn (P.X.); sunwenhui123@mail.dlut.edu.cn (W.S.); puzhongji@mail.dlut.edu.cn (Z.P.); 2 School of Food Science and Technology, Dalian Polytechnic University, Dalian 116034, China; liuyang_vsw@126.com; 3 College of Food Science and Engineering, Northwest A&F University, Yangling 712100, China; yaosiyu@nwafu.edu.cn; 4 School of Food and Environmental Science and Technology, Dalian University of Technology, Panjin124221, China

**Keywords:** *Fusarium proliferatum*, tomato plants, effector, pathogenicity, DEGs

## Abstract

Leaf spot disease caused by the fungus *Fusarium proliferatum* (Matsushima) Nirenberg is a destructive disease of tomato plants in China. Typical symptoms of infected tomato plants are softened and wilted stems and leaves, leading to the eventual death of the entire plant. In this study, we resorted to transcriptional profile analysis to gain insight into the repertoire of effectors involved in *F. proliferatum*–tomato interactions. A total of 61,544,598 clean reads were *de novo* assembled to provide a *F. proliferatum* reference transcriptome. From these, 75,044 unigenes were obtained, with 19.46% of the unigenes being assigned to 276 Kyoto Encyclopedia of Genes and Genomes (KEGG) pathways, with 22.3% having a homology with genes from *F. fujikuroi*. A total of 18,075 differentially expressed genes (DEGs) were identified, 720 of which were found to code for secreted proteins. Of these, 184 were identified as candidate effectors, while 79.89% had an upregulated expression. Moreover, 17 genes that were differentially expressed in RNA-seq studies were randomly selected for validation by quantitative real-time polymerase chain reaction (qRT–PCR). The study demonstrates that transcriptome analysis could be an effective method for identifying the repertoire of candidate effectors and may provide an invaluable resource for future functional analyses of *F. proliferatum* pathogenicity in *F. proliferatum* and tomato plant–host interactions.

## 1. Introduction

The tomato (*Solanum lycopersicum*) is one of the most important crop plants and one that also servesas a model system for fruit development [1]. According to the Food and Agriculture Organization Statistical Database (http://faostat3.fao.org), China was the largest tomato producer in 2013, with a total output of 50 million tons [2]. However, Chinese tomato production is threatened by diseases. Among these is tomato leaf spot caused by *Fusarium proliferatum* (Matsushima) Nirenberg, one of the most destructive fungal diseases for tomatoes [3]. The typical symptoms of tomato leaf spot on infected leaves and stems are necrotic spots that have a dark brown appearance and may continue to grow, causing the stems to soften and wilt, eventually leading to the death of the entire plant [3].

*Fusarium proliferatum*, a broadly distributed saprophytic pathogen, can cause destructive diseases to an extremely wide range of hosts that span several plant families, including maize [4], tomato [3], garlic [5] and soybean [6]. Additionally, *F. proliferatum* has been reported to produce a number of mycotoxins (including fusaric acid, fumonisin, fusaproliferin, beauvericin and moniliformin) that pose a serious threat to global food security and human health [7,8,9,10]. *Fusarium proliferatum* is an endophyte that dwells on the plant and produces a large number of conidia that can survive for many years in the soil [11]. As the weather becomes wetter and warmer, the conidia germinate and spread via atmospheric dust and rainwater movement. As a result of this, they end up infecting seeds, soil, as well as other plant materials. The germinated conidia enter the tomato plants through the stomata, and they form hyphae that grow along the vascular tissues and extend into the leaves and stem. Subsequently, the mycelia colonize the tomato plants, and this results in the appearance of leaf spots. As of now, the *F. oxysporum* infection process in tomatoes has been well documented [12,13,14], but *F. proliferatum*’s infection process on tomatoes has not been as thoroughly described. This is therefore an important problem that needs urgent solving.

At present, fungicides are the main management strategy for controlling fungal tomato diseases, but there is a lack of research on the response of *F. proliferatum* to different fungicides. Additionally, tomato cultivars resistant to tomato leaf spot are currently unavailable in the conventional market. Even if these management strategies were available, they would face a huge challenge due to the genetic variability of *F. proliferatum* rapidly emerging in the population [15].

Numerous genes of the filamentous plant pathogens have been shown to undergo diversified selection during the host–pathogen interaction [16]. More recently, it has been recognized that evolution has equipped *Fusarium* plant pathogens with a diverse range of infection strategies. These include the production and secretion of proteins and other molecules, collectively known as effectors, that successfully facilitate the infection process by reprogramming the host metabolism and by manipulating the immune responses of host cells to enable parasitic colonization [16,17]. Effectors are active outside the fungal cell and alter the host-cell structure and its function in order to generally facilitate the fungal lifestyle inside the plant and enhance access to nutrients [18]. Fungal effectors that trigger resistance or susceptibility in specific host plants have been identified in a number of ascomycetes that include *Fusarium* species. Most recent molecular studies of *Fusarium* pathogens have focused on investigating the secreted effectors produced by the pathogens during infection. Here, the use of transcriptomic analysis has helped identify secreted effectors in various *Fusarium* pathogens, such as the *F. oxysporum* species complex, *F. graminearum*, *F. verticillioides* and *F. virguliforme* [13,19,20,21]. For example, several effector genes secreted in xylem (*SIX*) were found during the *F. oxysporum,* f. sp. *Lycopersici*/tomato interaction, some of which were shown to be essential for pathogenicity [12,13,14]. Using transcriptional analysis, Lu and Edwards [19] have identified a list of potential small, secreted cysteine-rich protein-derived effectors produced by *F. graminearum* in the course of *F. graminearum*–wheat interaction. Brown et al. [20] indicated that secreted gene expression (*SGE1*) was required for pathogenicity and can affect synthesis of multiple secondary metabolites. *SGE1* has a role in the global regulation of transcription in *F. verticillioides*. Unraveling the secreted effectors of *F. proliferatum* produced during pathogenesis is therefore important to improve the control strategies for this pathogen.

The *F. proliferatum* genome has still not been sequenced, but genome sequences of several *Fusarium* species (*F. oxysporum*, *F. fujiluroi* and *F. graminearum*) have been deposited in the National Center of Biotechnology Information (NCBI) database. These can provide a rich trove of reference information from which to build an effective strategy for gaining more insight into effectors secreted in the *F. proliferatum*–tomato interaction [22,23,24]. Next-generation sequencing technologies have advanced rapidly, with ribonucleic acid sequencing (RNA-seq) becoming an instrumental assay for the analysis of fungal transcriptomes. By combing these with bioinformatics tools, putative secreted proteins can be predicted; of these, some that are relatively small (fewer than 200 amino acids) and contain a high percentage of cysteine residues (usually 2% to 20%) have been considered as effector molecules [18].

As of now, *F. proliferatum*’s infection process on tomatoes has not yet been as thoroughly described. In this work, we characterized the infection process of *F. proliferatum* in tomatoes in order to better understand the molecular basis ofthe *F. proliferatum*–tomato interaction. We then performed a *de novo* transcriptome analysis to predict putative effectors that may be contributing to the pathogenicity of *F. proliferatum* in tomatoes.

## 2. Results and Discussion

### 2.1. Characterization of Time Course of F. proliferatum-Infected Tomato Leaves

**Figure 1:**
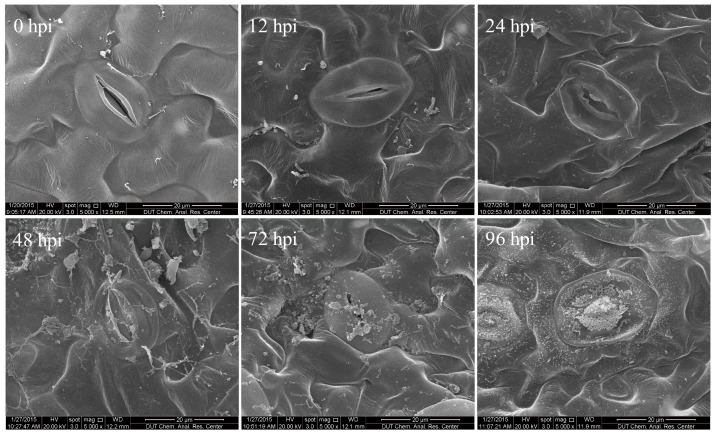
Preparation of *Fusarium proliferatum*-infected tomato leaves (*Solanum lycopersicum*) for transcriptome analysis. Leaves were obtained 0, 12, 24, 48, 72 and 96 h after inoculation (hpi) with a conidial suspension of *F. proliferatum*.

In order to obtain an overview of the *F. proliferatum* transcriptome and effector gene activity during the different phases of infection, we used a scanning electron microscope to observe six samples of *F. proliferatum*-infected tomato leaves at different infection time points (0, 12, 24, 48, 72 and 96 h after *F. proliferatum* inoculation) (Figure 1). Irregular epidermal cells were found on these leaves and the extent of this irregularity grew with a longer infection time. By 48 h post-inoculation (hpi), the stomata were invaded by the spores (Figure 1), and by 72 hpi, the number of spores on the leaf surface increased, accompanied by a corresponding increase in the number of invading spores on the stomata. By 96 hpi, the stomata and epidermal cells of the tomato leaves were covered with spores, and the leaves’s epidermal cells became more irregular and wrinkled. At the same time, the hyphae showed obvious growth on the stomata (Figure S1). Nguyen et al. [11] also showed that infection of maize leaf tissues by *F. proliferatum* occurred via the stomata, and that the microconidia of *F. proliferatum* that formed inside the leaf tissues sporulated through the stomata, which may provide nutrients to the pathogen. Based on this data, mycelia collected from 7-day-old potato dextrose agar (PDA) cultures (KC) and samples of infected leaf tissues taken 96 hpi (KS_1) were subjected to an RNA-seq analysis to reveal more candidate effector genes involved in *F. proliferatum*–tomato interactions.

### 2.2. De novo Assembly of F. proliferatum Transcriptome

By using RNA-seq technology, two transcriptomic datasets were generated from KC and KS_1 in order to better understand the pathogenicity of *F. proliferatum*. We obtained a total of 14.16 Gb of sequencing data, including 115,988,950 raw reads and 98,705,420 clean reads with a base average error rate below 0.03%. An overview of the transcriptome assembly statistics is shown in Table 1. After removing low-quality and adapter sequences, 37,091,012 and 61,614,408 clean reads were obtained for KC and KS_1 samples, respectively. The Q20 percentage and Q30 percentages were more than 96% and 92%, respectively. The average GC percentages in the KC and KS_1 samples were 51.58% and 53.15%, respectively.

**Figure 2:**
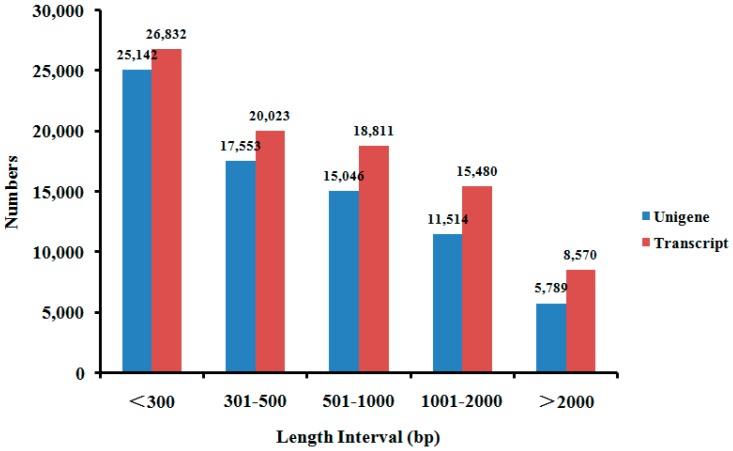
Distribution of transcript and unigene length in the assembled *F. proliferatum* reference transcriptome.

To establish the *F. proliferatum* transcriptome in the absence of a reference genome, the clean reads of KS_1 were mapped against the tomato genome [25]. A total of 24,453,586 clean reads did not map to the tomato genome. The clean reads of KC were consequently further pooled, yielding a total number of 61,544,598 reads, which were then used to perform the Trinity program for the *de novo* assembly of the *F. proliferatum* reference transcriptome. This analysis yielded 89,716 transcripts expressed from 75,044 unigenes (Table 2). The length of the unigenes ranged from 201 to 17,632 bp, with an N50 length of 1283 bp, a mean length of 767 bp and a median length of 419 bp. The transcript and unigene length distribution is shown in Figure 2. Around 26.80% of the transcripts were longer than 1 kb. To assess the quality of the sequencing and *de novo* assembly, all the assembled clean reads were mapped onto the *F. proliferatum* reference transcriptome. Mapping ratios of 93.31% and 74.65% were obtained for KC and KS_1, respectively.

### 2.3. F. proliferatum Reference Transcriptome Annotation

**Figure 3:**
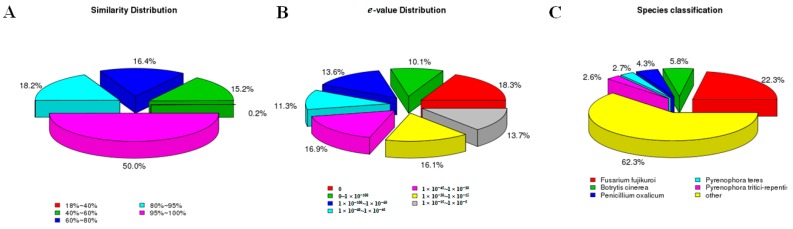
Species distribution of *F. Proliferatum* unigenes. (**A**) Similarity distribution of top Basic Local Alignment Search Tool （BLAST）hits for each unigene; (**B**) *e*-value distribution of BLAST hits with a cut off *e*-value of 1 × 10^−5^; (**C**) Species distribution for top BLAST hits in the Nr database.

In a search against the NCBI non-redundant (Nr) protein database, the annotation of the *de novo* assembled gene annotation revealed 46,292 unigenes (61.68%) with significant homology hits (*e*-value = 1 × 10^−5^) (Table 3). Half of the unigene sequences were more than 95% identical to the mapped sequences in the Nr database, while 70.2% of the unigenes had significant homology hits (*e*-value < 1 × 10^−30^) (Figure 3A, B). A total of 39,854 unigenes (53.10%) were matched to the SwissPort database. The mapping rates of the unigenes against the NCBI nucleotide (Nt), Kyoto Encyclopedia of Genes and Genomes Orthology (KO), Protein family (Pfam) and euKaryotic Ortholog Groups (KOG) databases were 83.30%, 28.21%, 50.09% and 32.43%, respectively. As shown in Table 3, a total of 71,878 unigenes (95.78%) were annotated in at least one database. According to a species classification analysis, only 16,734 unigenes (22.3%) had a high homology with the *Fusarium fujikuroi* genes, followed by the *Botrytis cinerea* (4352 unigenes, 5.8%) and *Penicillium oxalicum* (3226 unigenes, 4.3%) genes, while 46,752 unigenes (62.3%) had a high homology with sequences from other organisms (Figure 3C). According to BLASTx results, half of the *F. proliferatum* unigenes did not have any annotation, even though some of them were highly expressed. These unigenes may code for new proteins, which would account for the fact that no homologous genes from other *Fusarium* species could be found in the databases used this study.

The gene annotation showed that a total of 38,947 unigenes (51.89%) had at least one annotation characterized by gene ontology (GO) terms. According to the three Blast2GO categories, the GO terms of *F. proliferatum* unigenes could be grouped into the following categories: biological process, molecular function and cellular component (Figure S2). As shown in Table S1, the unigenes were assigned with one or more GO terms. In the biological process category, unigenes annotated to cellular process (18.14%) and single-organism process (14.02%) terms were the most dominant, followed by metabolic process (13.92%) and regulation of biological process (10.02%) terms. In the cellular component category, unigenes assigned to cell part (22.13%), membrane (16.12%) and membrane part (12.02%) terms were highly represented. Some of the unigenes were assigned to cell part terms, as a result of being of a similar category to the recently reported transcriptomes of *Fusarium oxysporum* f. sp. *Ciceris*, the fungus that causes vascular wilt in chickpeas [26]. For the molecular function category, unigenes related to catalytic activity (37.65%) and binding (32.71%) were found to be most abundant.

**Figure 4:**
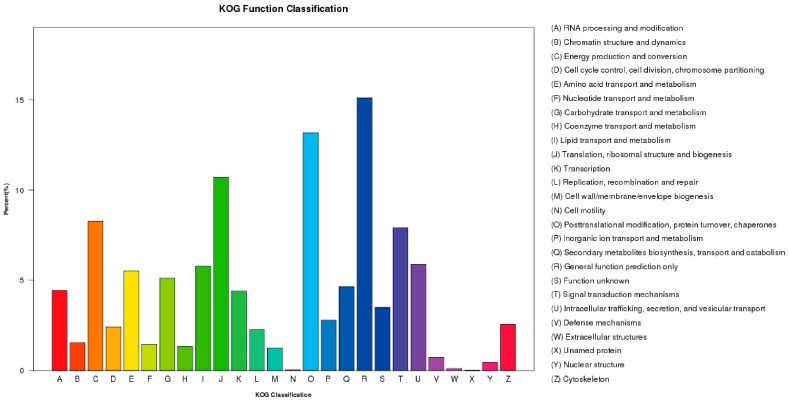
euKaryotic Ortholog Groups (KOG) functional categories for *F. proliferatum*.

After the GO analysis, the unigenes were assigned to the KOG database for a functional prediction and classification. A total of 24,338 unigenes were grouped into 26 KOG classifications (Figure 4). A high percentage of unigenes was assigned to the KOG’s general function prediction (3674, 15.09%) category, followed by the following categories: posttranslational modification, protein turnover, chaperones (3206, 13.17%), translation, ribosomal structure and biogenesis (2604, 10.69%), energy production and conversion (2014, 8.27%), and signal transduction mechanisms (1924, 7.90%).

**Figure 5:**
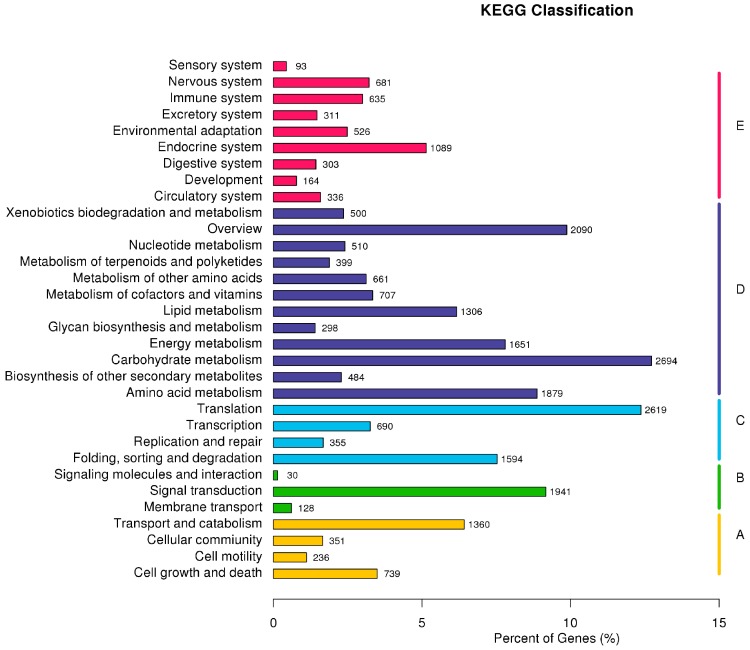
Pathway assignment based on the Kyoto Encyclopedia of Genes and Genomes (KEGG). (**A**) Classification based on cellular processes categories; (**B**) classification based on environmental-information processing categories; (**C**) classification based on genetic-information processing categories; (**D**) classification based on metabolism categories; and (**E**) classification based on organismal-system categories.

Meanwhile, the Kyoto Encyclopedia of Genes and Genomes (KEGG) database was used to search for active biochemical pathways in all unigenes of *F. proliferatum*. A total of 14,607 unigenes were clustered into 276 KEGG pathways (Figure 5 and Table S2), and the most represented classification was based on metabolism categories. Carbohydrate metabolism (2694 unigenes), amino acid metabolism (1879 unigenes), energy metabolism (1651 unigenes) and lipid metabolism (1306 unigenes) were the main metabolic pathways. KEGG organismal-system categories included the endocrine system (1089 unigenes), nervous system (681 unigenes) and immune system (635 unigenes). In the genetic-information processing categories, the most significant enriched KEGG pathways were translation (2619 unigenes), followed by folding, sorting and degradation (1594 unigenes). Additionally, the most abundant subcategories in KEGG environmental-information processing and cellular processes categories were signal transduction (1941 unigenes) and transport and catabolism (1360 unigenes), respectively. The above functional annotations indicated that the clustered unigenes represented an extensive catalog encompassing a large proportion of the genes expressed in *F. proliferatum*.

### 2.4. F. proliferatum Differental Gene Expression in KS_1

After mapping against the *de novo F. proliferatum* transcriptome, a total of 18,075 differentially expressed genes (DEGs) were found to display a significant differential expression in KS_1 compared to KC (*q*-value < 0.005 and |log_2_ (foldchange)| > 1) (Figure S3). Overall, the majority of the DEGs (14,766, 81.69%) in KS_1 were upregulated genes, while only 3309 DEGs were downregulated genes, suggesting a strong interaction between *F. proliferatum* and the tomato leaves.

**Figure 6:**
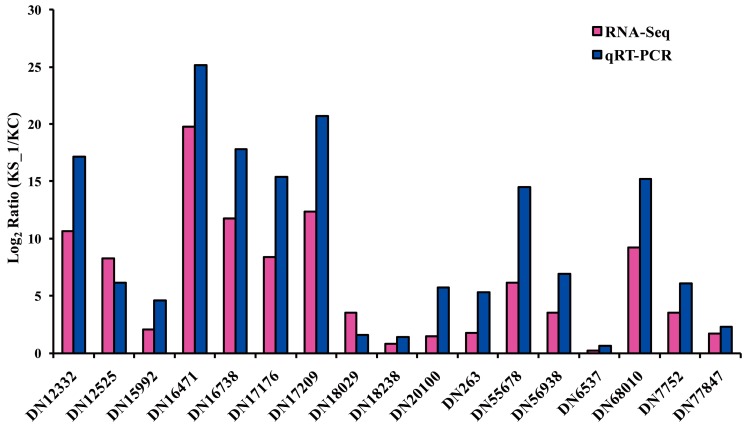
Validation of the expression of differentially expressed genes (DEGs) in *F. proliferatum* by Quantitative real-time PCR (qRT-PCR).

Our transcriptome analysis provides a further view on the expression of *F. proliferatum* genes at 96 hpi. However, it is worth pointing out that our RNA-seq analyses were generated from two treatments, one from KS_1 and the other from KC. As a consequence, the interpretation of the transcriptome data may be potentially biased. To confirm the RNA-seq profiles, quantitative real-time PCR (qRT–PCR) was used to examine 17 randomly selected genes using three independent biological replicates of KS_1 and KC (Figure 6). The fold change (log_2_ ratio) was used to validate the results when comparing qRT–PCR gene-expression levels with the RNA-seq gene FPKM (fragments per kilobase of exon per million fragments mapped) values (Table S3). The result indicated that the unigenes’ expression profiles obtained from the two approaches were basically consistent.

Using the *F. proliferatum* reference transcriptome, a GO enrichment analysis compared KS_1 and KC in order to understand the functional differences among DEGs. A corrected *p*-value below 0.05 indicated that the function was enriched. In the GO enrichment analysis, the biological process category was most abundant, followed by the molecular function category and the cellular component category (Figure S4). Highly enriched DEGs in KS_1were involved in the macromolecular complex, hydrolase activity, protein complex, ion transport and substrate-specific transporter activity. The macromolecular complex has been shown to be involved in the catabolic process of chitin, which is usually associated with the biosynthesis of fungal cell walls [26]. Hydrolase activity, released during the infection process, is key to the maintenance of wall plasticity associated with the fungal cell [27]. Our data indicated that the genes for ion transport were significantly enriched in the enriched DEGs in KS_1, suggesting that these DEGs may be essential for the pathogen to absorb nutrients from its host and for it to export fungal secondary metabolites and toxic compounds to the outside.

The KEGG enrichment analysis also compared KS_1 and KC in order to elucidate the significantly enriched biochemical pathway of DEGs in *F. proliferatum* (Figure S5). The most upregulated DEGs in KS_1 were those that were involved in the oxidative phosphorylation pathway, followed by the starch and sucrose metabolism (Figure S6A). Other upregulated DEGs in KS_1 included those associated with protein processing in the endoplasmic reticulum, ribosome, endocytosis, and amino sugar and nucleotide sugar metabolism. The major pathways triggered by *F. oxysporum*. f. sp. *ciceris* during conidial germination include the starch and sucrose metabolism, the amino sugar and nucleotide sugar metabolism, and the propanoate metabolism [26]. We also found these pathways in our study, and a large number of upregulated DEGs were associated with them. However, most of the DEGs in KS_1 involved in RNA transport, the cysteine and methionine metabolism, various types of *N*-glycan biosyntheses, lysine degradation, the purine metabolism and RNA degradation were upregulated (Figure S6B).

### 2.5. F. proliferatum Candidate Effectors

In plant–microbe interactions, some secreted proteins play an important role in promoting the fungal infection of host plants. Filamentous pathogens are known to secrete an arsenal of effector proteins that regulate innate immunity in plants and that facilitate the development of plant diseases [28]. Numerous effectors of *Fusarium* have been identified, such as *F. oxysporum*, *F. graminearum*, *F. verticillioides* and *F. virguliforme* [16,18,19,20]. However, the effectors in *F. proliferatum* have not been well studied on the molecular level. A total of 184 candidate effector genes were identified from the DEGs that were used to encode secreted proteins (Table S4), and most of these candidate effector genes (147,79.89%) were upregulated in KS_1. The cysteine content of 184 candidate-effectors encoded proteins ranged from 2.02% to 15.31%, with 24 candidate effectors having more than 10% of cysteine, while the majority (128, 69.65%) had less than 5% cysteine. This result is similar to the cysteine content previously identified in small secreted cysteine-rich proteins in *F. graminearum* [18].

As anticipated, 39.13% of the candidate effectors in this study lacked homology with known proteins and were annotated as hypothetical proteins in the Nr database. This is consistent with the identified effectors from filamentous fungi [29,30]. Only 37 candidate effectors (20.10%) had functional annotations in the Nr database (Table S4). Further analysis revealed that some of the secreted proteins reportedly associated with fungal pathogenicity were also found in our study: for example, the glycosylphosphatidylinol (GPI)-anchored cysteine-rich fungal effector motif (CFEM) domain protein, the cell-wall protein, and the hydrophobin and blastomyces yeast-phase-specific (BYS1) domain protein. The CFEM domain is conserved in ascomycetes and is an inadenylate cyclase (MAC1)-interacting (ACI1) protein first discovered and isolated from *Magnaporthe grisea* [31]. CFEM-containing proteins play important roles in the pathogenesis of fungi, acting as signal transducers, cell-surface receptors, or adhesion molecules in host–pathogen interactions [31]. After BLAST, three candidate effectors (DN78913, DN18169 and DN67233) were found to contain the CFEM domain against the domain conserved in the Nr database. The functional annotation indicated that DN18169 and DN67233 may belong to the GPI-anchored CFEM domain protein. The GPI-anchored CFEM domain protein can interact with a fungal adenylate cyclase controlling appressorium formation, which is a critical step in the development of rice blast disease [31] and fusarium head blight (a devastating disease in wheat) [18]. Hydrophobins are secreted proteins that are expressed during plant–fungus interaction. In addition, they are located on the outer surfaces of the cell walls of the mycelia and conidia, and they have been shown to mediate fungus–host interaction [32]. We found that two candidate effectors (DN18029 and DN2243) are homologous to hydrophobins. The BYS1-domain protein was originally purified from the pathogenic dimorphic fungus *Blastomyces dermatitidis* [33], which was found in two candidate effectors (DN33323 and DN69307), suggesting that the BYS1-domain protein may be involved in the pathogenicity of *F. proliferatum*-infected tomato plants. On the other hand, concanavalin A-like lectin/glucanase has been identified as an effector protein in *Pyrenophorateres* f. *teres* [34], which was also found in the candidate effector DN57353. Candidate effectors homologous to acid phosphatase, adhesin, clock-controlled protein-like protein, glycoside hydrolase protein and tyrosinase were also identified from the secreted proteins of *F. proliferatum*-infected tomato plants.

**Figure 7:**
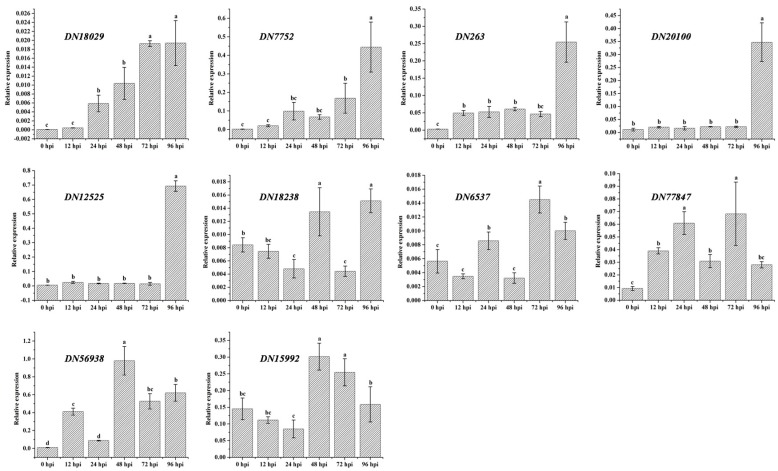
Expression levels of ten *F. proliferatum* candidate effector genes at six different time points (0, 12, 24, 48, 72 and 96 hpi) as determined by qRT–PCR analyses. The β-tubulin and ubiquitin genes were used as two internal control genes for normalization.

Finally, to investigate the expression profiles of the candidate effector genes at six different time points following infection during *F. proliferatum*–tomato interaction, ten of the 17 candidate effector genes confirmed above by qRT–PCR were subjected again to qRT–PCR using infected leaves from 0, 12, 24, 48, 72 and 96 hpi. The expression of DN18029 increased steadily from 0 to 96 hpi, but high expression levels of DN263, DN20100 and DN12525 were observed only at 96 hpi (Figure 7). However, DN7752 and DN18238 were expressed at relatively high levels at 96 hpi, whereas DN56938 and DN15992 were expressed at high levels at 48 hpi, and DN6537 and DN77847 at 72hpi.

## 3. Materials and Methods

### 3.1. Biological Material and Inoculation Assays

The *Fusarium proliferatum* (Matsushima) Nirenberg strain used in this study was originally isolated from the leaves and stems of infected tomato plants obtained from a commercial tomato greenhouse in Dalian, Liaoning Province, China in January 2014, and it was obtained as previously described [3]. Tomato plants (*S. lycopersicum* ′Zaofen No. 2′) were grown individually in plastic pots (10 cm diameter × 8 cm height) and placed in a growth chamber set at 25 °C under a 16 h light/8 h dark photoperiod. Mycelia and spores were harvested from the fungus grown on PDA medium at 25 °C in the dark for 7 days. To simulate a *F. proliferatum* infection, three-week-old tomato plants were sprayed with a conidial suspension of *F. proliferatum* containing 1 × 10^8^ spores/mL. After spraying, the plants were placed in a 25 °C dark growth chamber with 100% relative humidity for 24 h.

### 3.2. Evaluation of Time Course of F. proliferatum Infection

The inoculation assays of *F. proliferatum*-infected tomato plants were performed as described above. Infected leaf tissues were collected at 0, 12, 24, 48, 72 and 96 hpi after the *F. proliferatum* were sprayed with a conidial suspension containing 1 × 10^8^ spores/mL. The mycelia and infected leaf materials were immediately frozen in liquid nitrogen and stored at −80 °C. After the RNA extraction, the samples were subjected to RNA-seq sequencing and qRT-PCR to verify the observed gene expression patterns. For microscopic observation, *F. proliferatum*-infected tomato leaves were manually cut into 2 to 3 mm pieces with a sterile scalpel and immediately placed in a pre-cooled 2.5% glutaraldehyde fixative for at least 2 h at 4 °C at different time points. The samples were observed using the Nova NanoSEM450 scanning electron microscope (FEI Corporation, San Francisco, CA, USA). Our observations were carried out for leaves taken at different hpis.

### 3.3. Transcriptome Profiling

The tomato plants were inoculated with *F. proliferatum* as described previously. The *Fusarium proliferatum* (Matsushima) Nirenberg strain was cultured in a PDA medium at 25 °C in the dark for 7 days. The mycelia was then gently scraped from the plates with a sterile lab spoon as a KC sample. For total RNA extraction, mycelia and infected leaf materials were ground to a powder in liquid nitrogen using a mortar and pestle using TRIzol^®^ LS Reagent (Invitrogen, Carlsbad, CA, USA) following the manufacturer’s instructions. For the RNA-seq analysis, RNA isolation was conducted using mycelia collected from 7-day-old PDA cultures (KC), and infected leaf tissues taken 96 hpi (KS_1) after *F. proliferatum*’s inoculation via a conidial suspension. For each specimen, RNA was extracted from a mixture of three independent biological replicates. Polyadenylated (Poly(A)) mRNA was isolated from the total RNA, and cDNA libraries were then constructed and sequenced at the 2 × 150 bp paired-end read mode with Illumina HiSeq^®^2500, performed at Novogene Corporation, Beijing, China, in accordance with the manufacturer's standard protocol.

After discarding the adapter-only reads, subsequent analyses first used low-quality reads with ambiguous bases and reads with more than 50% Qphred ≤ 20 bases from generated raw paired-reads, then used the clean reads from the filtered and trimmed reads. The quality of the two libraries was controlled with FastQC. As the KS_1 sample contained both host and fungal transcriptomes and the KC sample contained only the fungal transcriptome, the clean reads from the KS_1 library were aligned with the *S. lycopersicum SL2.50* genome (https://www.ncbi.nlm.nih.gov/genome/?term=Solanum+lycopersicum+L), and the unmapped clean reads were generated by using TopHat version 2.0.6 [35]. Following this, the clean reads from the KC library and the unmapped clean reads from the KS_1 library were used as input to generate a preliminary assembly via the *de novo* assembly of the *F. proliferatum* transcriptome using Trinity software with a default *k*-mer length of 25 [36]. The assembled contigs were processed with CD–HIT–EST with an identity threshold of 95% in order to remove redundant transcripts [37]. Low-complexity sequences were masked using DustMasker, and sequences with fewer than 200 bp were discarded. After the assembly, a unigene dataset was produced by performing clustering on scaffolds using TGI Clustering tools [38]. Again, the unigenes were mapped to the *S. lycopersicum* genome to further exclude the contaminating sequences. Finally, the resulting transcript dataset was taken as a *F. proliferatum* reference transcriptome for further analysis.

### 3.4. Transcriptome Annotation and DEG

An open reading frames (ORFs) prediction was performed using the European molecular biology open software suite [39], and the longest ORF was extracted from each unigene. After this, all unigenes were annotated using BLASTx alignments by comparing their sequences with various protein databases, including the Nr (http://www.ncbi.nlm.nih.gov/), SwissPort (http://www.ebi.ac.uk/uniprot/), KOG (http://www.ncbi.nlm.nih.gov/KOG/) and KEGG (http://www.genome.jp/kegg/) databases, with an *e*-value cutoff of 1 × 10^−5^. A functional annotation by GO term of all the assembled unigenes was performed with the Blast2GO program (https://www.blast2go.com/). Finally, the WEGO software (http://wego.genomics.org.cn/) was used to perform a GO function classification and reveal the distribution of gene functions in *F. proliferatum* at the macromolecular level. Gene expression levels were estimated by RNA-seq expression estimation for the two samples by expectation maximization (RSEM) [40], and FPKM was the most commonly used method to normalize gene expression levels [41].

Prior to the differential gene expression analysis, the edgeR program package was used to adjust the read counts in each sequenced library. To identify DEGs, a comparison between the two samples was performed using the DEGseq 1.12.0 R package (R Foundation for Statistical Computing, Vienna, Austria) [42]. The *p*-value was adjusted using the *q*-value [43], and the fold change (log_2_ratio) was estimated according to the normalized gene expression level in the two samples. In this paper, *q*-value < 0.005 and |log_2_ (foldchange)| > 1 were set as the threshold for the DEGs.

### 3.5. Candidate Effector Gene Prediction

Several prediction algorithms were utilized to predict the putative secreted proteins. The program TargetP 1.1 (http://www.cbs.dtu.dk/services/TargetP/) was used to predict the cleavage sites for the predicted presequences. Signal peptide cleavage sites were identified using SignalP 4.1 (http://www.cbs.dtu.dk/services/SignalP/), after which transmembrane helices were detected with TMHMM 2.0 (http://www.cbs.dtu.dk/services/TMHMM-2.0/) [4]. Putative candidate effector proteins identified through the transcriptomic analysis of *F. proliferatum* were selected based on four conditions: (1) the presence of N-terminal signal peptide cleavage sites; (2) the effector proteins having fewer than 200 amino acids; (3) the percentage of cysteine content being greater or equal to 2%; and (4) no transmembrane helices in the mature proteins.

### 3.6. Quantitative RT-PCR Assay

The tomato plants were inoculated with *F. proliferatum* as described previously. Infected leaf tissues were collected at 0, 12, 24, 48, 72 and 96 hpi after the *F. proliferatum* was sprayed with a conidial suspension containing 1 × 10^8^ spores/mL. Following this, the total RNA of each sample was extracted as described previously. Seventeen of the DEGs identified via the RNA-seq analysis were randomly selected for confirmation by qRT–PCR. The primers were designed from ten candidate-effector gene sequences using Primer-Premier 5 software (Premier Biosoft Interpairs, Palo Alto, CA, USA). The β-tubulin [44] and ubiquitin [23] genes were both used simultaneously as two internal control genes in order to obtain a more accurate quantitative result (Table S5). For the qRT–PCR, a first-strand cDNA synthesis was performed using RNA samples from KC, KS_1 and the tomato leaf tissues collected at six different time points (0, 12, 24, 48, 72 and 96 hpi) following *F. proliferatum*’s inoculation via a conidial suspension. This synthesis was performed using a PrimeScript™ RT reagent kit with a gDNA Eraser (TaKara, Dalian, China), in accordance with the manufacturer's instruction. The Mx3005p™ detection system (Agilent Stratagene, Santa Clara, CA, USA) was used to determine gene expression viaqRT–PCR. Quantitative RT–PCR was performed on cDNA samples (diluted 1:10) using SYBR^®^*Premix Ex Taq*™ II (TliRNaseH Plus) (Takara), in accordance with the manufacturer’s instruction. The samples were first incubated at 95 °C for 30 s. This was followed by 40 amplification cycles at 95 °C for 10 s, 60 °C for 25 s, and then 72 °C for 25 s. Using the geNorm program manual, the threshold cycle (*CT*) values of two reference genes and ten candidate effector genes were quantified with the comparative *CT* Method. The normalization factor values of the two reference genes were then automatically calculated via geNorm. The standard deviation was calculated according to the mathematical formulae in the geNorm manual [45]. The data for KC, KS_1 and the tomato leaf tissues collected at six different time points were analyzed by one way-ANOVA contained in the SPSS 17.0 software. Differences between samples were considered to be statistically significant at the *p* < 0.05 level.

### 3.7. Accession Numbers

All RNA-seq reads generated in this study were deposited at the GenBank SRA database (http://www.ncbi.nlm.nih.gov/sra) under the BioProject ID PRJNA397359.

## 4. Conclusions

In conclusion, this study is the first to report the use of transcriptome analysis as a means of screening effector genes that are involved in *F. proliferatum*-infected tomato interactions. *De novo* sequencing of the *F. Proliferatum* transcriptome yielded new insights into the molecular pathogenicity of this important tomato plant fungus. Using bioinformatics and functional analysis, a total of 184 candidate effector genes were identified from a high number of DEGs. Most of the candidate effector genes were expressed as hypothetical proteins, so the functional verification of these candidate effector genes and their respective roles in *F. proliferatum* would need further investigation. There is no doubt that the result of such findings will accelerate the identification of effector genes that play a key role in the resistant or susceptible responses of the tomato plants.

## Figures and Tables

**Table 1:** Summary of the RNA-Seq data.

cDNA Library	Raw Reads	Clean Reads	Clean Bases (Gb)	Error (%)	Q20 (%) ^1^	Q30 (%) ^2^	GC (%)
KC	52,509,182	37,091,012	6.46	0.03	96.28	92.16	51.58
KS_1	63,479,768	61,614,408	7.70	0.03	96.13	92.29	53.15

^1^ Q20: percentage of bases with a Phred value >20; ^2^ Q30: percentage of bases with a Phred value >30.

**Table 2:** Sequence summary of transcriptome assembly statistics.

Category	Total Number	Min Length (bp)	Mean Length (bp)	Median Length (bp)	Max Length (bp)	N50
Transcripts	89,716	201	853	471	17,632	1443
Unigenes	75,044	201	767	419	17,632	1283

**Table 3:** Annotationresults of the assembled unigenes.

Database	Number of Unigenes	Percentage (%)
Annotated in Nr	46,292	61.68
Annotated in Nt	62,514	83.3
Annotated in KO	21,172	28.21
Annotated in SwissPort	39,854	53.10
Annotated in Pfam	37,595	50.09
Annotated in GO	38,947	51.89
Annotated in KOG	24,338	32.43
Annotated in all databases	12,073	16.08
Annotated in at least one database	71,787	95.78
Total unigenes	75,044	100

KO: Kyoto Encyclopedia of Genes and Genomes Orthology; GO: Gene ontology; KGO: euKaryotic Ortholog Groups.

## Supplementary Materials

Supplementary materials can be found at www.mdpi.com/1422-0067/19/1/31/s1.

## Abbreviations

DEGs	Differentially expressed genes
qRT–PCR	Quantitative real-time PCR
*SIX*	Secreted in xylem
*SGE1*	Secreted gene expression
NCBI	National Center of Biotechnology Information
RNA-seq	Ribonucleic acid sequencing
Hpi	Hourspost-inoculation
PDA	Potato dextrose agar
Nr	Non-redundant
Nt	Nucleotide
KEGG	Kyoto encyclopedia of genes and genomes
KO	Kyoto encyclopedia of genes and genomes Orthology
Pfam	Protein family
KOG	euKaryotic Ortholog Groups
BLAST	Basic Local Alignment Search Tool
GO	Gene ontology
FPKM	Fragments per kilobase of exon per million fragments mapped
GPI	Glycosylphosphatidylinol
CFEM	Cysteine-rich fungal effector motif
BYS1	Blastomyces yeast-phase-specific
Poly(A)	Polyadenylated (Poly(A))
ORFs	Open reading frames
RSEM	RNA-seq expression estimation by expectationmaximization
*CT*	Threshold cycle

## References

[1] Consortium T.T.G. (2012). The tomato genome sequence provides insights into fleshy fruit evolution. Nature.

[2] Suwannarach N., Kumla J., Nitiyon S., Limtong S., Lumyong S. (2016). First report of sour rot on tomato caused by *Galactomyces reessii* in Thailand. J. Gen. Plant Pathol..

[3] Gao M.L., Luan Y.S., Yu H.N., Bao Y.M. (2016). First report of tomato leaf spot caused by *Fusarium proliferatum* in China. Can. J. Plant Pathol..

[4] Peltomaa R., Vaghini S., Patiño B., Benito-Peña E., Moreno-Bondi M.C. (2016). Species-specific optical genosensors for the detection of mycotoxigenic *Fusarium* fungi in food samples. Anal. Chim. Acta.

[5] Seefelder W., Gossmann M., Humpf H.U. (2002). Analysis of Fumonisin B1 in *Fusarium proliferatum*-Infected Asparagus Spears and Garlic Bulbs from Germany by Liquid Chromatography−Electrospray Ionization Mass Spectrometry. J. Agric. Food Chem..

[6] Chang K.F., Hwang S.F., Conner R.L., Ahmed H.U., Zhou Q., Turnbull G.D., Strelkov S.E., Mclaren D.L., Gossen B.D. (2015). First report of *Fusarium proliferatum* causing root rot in soybean (*Glycine max* L.) in Canada. Crop Prot..

[7] Palmero D., De Cara M., Nosir W., Galvez L., Cruz A., Woodward S., Gonzalez-Jaen M.T., Tello J.C. (2012). *Fusarium proliferatum* isolated from garlic in Spain: Identification, toxigenic potential and pathogenicity on related *Allium* species. Phytopathol. Mediterr..

[8] Rheeder J.P., Marasas W.F.O., Vismer H.F. (2002). Production of Fumonisin Analogs by *Fusarium* Species. Appl. Environ. Microbiol..

[9] Gil-Serna J., Gálvez L., París M., Palmero D. (2016). *Fusarium proliferatum* from rainwater and rooted garlic show genetic and pathogenicity differences. Eur. J. Plant Pathol..

[10] Isack Y., Benichis M., Gillet D., Gamliel A. (2014). A selective agar medium for isolation, enumeration and morphological identification of *Fusarium proliferatum*. Phytoparasitica.

[11] Nguyen T.T., Dehne H.W., Steiner U. (2016). Histopathological assessment of the infection of maize leaves by *Fusarium graminearum*, *F. proliferatum*, and *F. verticillioides*. Fungal Biol..

[12] Taylor A., Vágány V., Jackson A.C., Harrison R.J., Rainoni A., Clarkson J.P. (2016). Identification of pathogenicity-related genes in *Fusarium oxysporum* f. sp. *cepae*. Mol. Plant Pathol..

[13] Rocha L.O., Laurence M.H., Ludowici V.A., Puno V.I., Lim C.C., Tesoriero L.A., Summerell B.A., Liew E.C.Y. (2016). Putative effector genes detected in *Fusarium oxysporum* from natural ecosystems of Australia. Plant Pathol..

[14] Rep M., van der Does H.C., Meijer M., van Wijk R., Houterman P., Dekker H., de Koster C., Cornelissen B. (2010). A small, cysteine-rich protein secreted by *Fusarium oxysporum* during colonization of xylem vessels is required for *I-3*-mediated resistance in tomato. Mol. Microbiol..

[15] Dong Y., Li Y., Zhao M., Jing M., Liu X., Liu M., Guo X., Zhang X., Chen Y., Liu Y. (2015). Global Genome and Transcriptome Analyses of *Magnaporthe oryzae* Epidemic Isolate 98-06 Uncover Novel Effectors and Pathogenicity-Related Genes, Revealing Gene Gain and Lose Dynamics in Genome Evolution. PLoS Pathog..

[16] Sperschneider J., Gardiner D.M., Thatcher L.F., Lyons R., Singh K.B., Manners J.M., Taylor J.M. (2015). Genome-Wide Analysis in Three *Fusarium* Pathogens Identifies Rapidly Evolving Chromosomes and Genes Associated with Pathogenicity. Genome Biol. Evol..

[17] Hogenhout S.A., van der Hoorn R.A., Terauchi R., Kamoun S. (2009). Emerging concepts in effector biology of plant-associated organisms. Mol. Plant Microbe Interact..

[18] Win J., Chaparro-Garcia A., Belhaj K., Saunders D.G., Yoshida K., Dong S., Schornack S., Zipfel C., Robatzek S., Hogenhout S.A. (2012). Effector biology of plant-associated organisms: Concepts and perspectives. Cold Spring Harb. Symp. Quant. Biol..

[19] Lu S., Edwards M.C. (2016). Genome-Wide Analysis of Small Secreted Cysteine-Rich Proteins Identifies Candidate Effector Proteins Potentially Involved in *Fusarium graminearum*-Wheat Interactions. Phytopathology.

[20] Brown D.W., Busman M., Proctor R.H. (2014). *Fusarium verticillioidesSGE1* is required for full virulence and regulates expression of protein effector and secondary metabolite biosynthetic genes. Mol. Plant Microbe Interact..

[21] Chang H.X., Domier L., Radwan O., Yendrek C., Hudson M., Hartman G.L. (2016). Identification of multiple phytotoxins produced by *Fusarium virguliforme* including a phytotoxic effector (FvNIS1) associated with sudden death syndrome foliar symptoms. Mol. Plant Microbe Interact..

[22] Ma L.J., Shea T., Young S., Zeng Q., Kistler H.C. (2014). Genome Sequence of *Fusarium oxysporum* f. sp. *melonis* Strain NRRL 26406, a Fungus Causing Wilt Disease on Melon. Genome Announc..

[23] Wiemann P., Sieber C.M.K., Bargen K.W.V., Studt L., Niehaus E.M., Espino J.J., Huß K., Michielse C.B., Albermann S., Wagner D. (2013). Deciphering the Cryptic Genome: Genome-wide Analyses of the Rice Pathogen *Fusarium fujikuroi* Reveal Complex Regulation of Secondary Metabolism and Novel Metabolites. PLoS Pathog..

[24] Gardiner D.M., Stiller J., Kazan K. (2014). Genome Sequence of *Fusarium graminearum* Isolate CS3005. Genome Announc..

[25] Aoki K., Yano K., Suzuki A., Kawamura S., Sakurai N., Suda K., Kurabayashi A., Suzuki T., Tsugane T., Watanabe M. (2010). Large-scale analysis of full-length cDNAs from the tomato (*Solanum lycopersicum*) cultivar Micro-Tom, a reference system for the Solanaceae genomics. BMC Genom..

[26] Sharma M., Sengupta A., Ghosh R., Agarwal G., Tarafdar A., Nagavardhini A., Pande S., Varshney R.K. (2016). Genome wide transcriptome profiling of *Fusarium oxysporum* f sp. *ciceris* conidial germination reveals new insights into infection-related genes. Sci. Rep..

[27] Adams D.J. (2004). Fungal cell wall chitinases and glucanases. Microbiology.

[28] Kamoun S. (2007). Groovy times: Filamentous pathogen effectors revealed. Curr. Opin. Plant Biol..

[29] Ellis J.G., Rafiqi M., Gan P., Chakrabarti A., Dodds P.N. (2009). Recent progress in discovery and functional analysis of effector proteins of fungal and oomycete plant pathogens. Curr. Opin. Plant Biol..

[30] Stergiopoulos I., de Wit P.J. (2009). Fungal Effector Proteins. Annu. Rev. Phytopathol..

[31] Kulkarni R.D., Kelkar H.S., Dean R.A. (2003). An eight-cysteine-containing CFEM domain unique to a group of fungal membrane proteins. Trends Biochem. Sci..

[32] Guzmán-Guzmán P., Alemán-Duarte M.I., Delaye L., Herrera-Estrella A., Olmedo-Monfil V. (2017). Identification of effector-like proteins in *Trichoderma* spp. and role of a hydrophobin in the plant-fungus interaction and mycoparasitism. BMC Genet..

[33] Burg E.F., Smith L.H. (1994). Cloning and characterization of *bys1*, a temperature-dependent cDNA specific to the yeast phase of the pathogenic dimorphic fungus *Blastomyces dermatitidis*. Infect. Immun..

[34] Ismail I.A., Able A.J. (2016). Secretome analysis of virulent *Pyrenophora teres* f. *teres* isolates. Proteomics.

[35] Trapnell C., Pachter L., Salzberg S.L. (2009). TopHat: Discovering splice junctions with RNA-Seq. Bioinformatics.

[36] Grabherr M.G., Haas B.J., Yassour M., Levin J.Z., Thompson D.A., Amit I., Adiconis X., Fan L., Raychowdhury R., Zeng Q. (2011). Full-length transcriptome assembly from RNA-Seq data without a reference genome. Nat. Biotechnol..

[37] Li W., Godzik A. (2006). Cd-hit: A fast program for clustering and comparing large sets of protein or nucleotide sequences. Bioinformatics.

[38] Pertea G., Huang X., Liang F., Antonescu V., Sultana R., Karamycheva S., Lee Y., White J., Cheung F., Parvizi B. (2003). TIGR Gene Indices clustering tools (TGICL): A software system for fast clustering of large EST datasets. Bioinformatics.

[39] Rice P., Longden I., Bleasby A. (2000). EMBOSS: The European Molecular Biology Open Software Suite. Trends Genet..

[40] Li B., Dewey C.N. (2011). RSEM: Accurate transcript quantification from RNA-Seq data with or without a reference genome. BMC Bioinform..

[41] Trapnell C., Williams B.A., Pertea G., Mortazavi A., Kwan G., van Baren M.J., Salzberg S.L., Wold B.J., Pachter L. (2010). Transcript assembly and quantification by RNA-Seq reveals unannotated transcripts and isoform switching during cell differentiation. Nat. Biotechnol..

[42] Wang L., Feng Z., Wang X., Wang X., Zhang X. (2010). DEGseq: An R package for identifying differentially expressed genes from RNA-seq data. Bioinformatics.

[43] Storey J.D. (2003). The Positive False Discovery Rate: A Bayesian Interpretation and the *q*-Value. Ann. Stat..

[44] Thatcher L.F., Gardiner D.M., Kazan K., Manners J.M. (2011). A highly conserved effector in *Fusarium oxysporum* is required for full virulence on *Arabidopsis*. Mol. Plant Microbe Interact..

[45] Vandesompele J., Preter K.D., Pattyn F., Poppe B., Roy N.V., Paepe A.D., Speleman F. (2002). Accurate normalization of real-time quantitative RT-PCR data by geometric averaging of multiple internal control genes. Genome Biol..

